# Melanin-Concentrating Hormone (MCH): Role in Mediating Reward-Motivated and Emotional Behavior and the Behavioral Disturbances Produced by Repeated Exposure to Reward Substances

**DOI:** 10.3390/ijms26157143

**Published:** 2025-07-24

**Authors:** Olga Karatayev, Sarah F. Leibowitz

**Affiliations:** Laboratory of Behavioral Neurobiology, The Rockefeller University, New York, NY 10065, USA; karatao@rockefeller.edu

**Keywords:** melanin-concentrating hormone (MCH), hypocretin/orexin (HCRT), molecular mechanisms, prenatal exposure to rewarding substances, alcohol, nicotine, cocaine, palatable fat-rich food, neurodevelopment, substance use disorders, neurological conditions

## Abstract

Clinical and animal studies suggest that multiple brain systems are involved in mediating reward-motivated and related emotional behavior including the consumption of commonly used drugs and palatable food, and there is evidence that the repeated ingestion of or exposure to these rewarding substances may in turn stimulate these brain systems to produce an overconsumption of these substances along with co-occurring emotional disturbances. To understand this positive feedback loop, this review focuses on a specific population of hypothalamic peptide neurons expressing melanin-concentrating hormone (MCH), which are positively related to dopamine reward and project to forebrain areas that mediate this behavior. It also examines neurons expressing the peptide hypocretin/orexin (HCRT) that are anatomically and functionally linked to MCH neurons and the molecular systems within these peptide neurons that stimulate their development and ultimately affect behavior. This report first describes evidence in animals that exposure in adults and during adolescence to rewarding substances, such as the drugs alcohol, nicotine and cocaine and palatable fat-rich food, stimulates the expression of MCH as well as HCRT and their intracellular molecular systems. It also increases reward-seeking and emotional behavior, leading to excess consumption and abuse of these substances and neurological conditions, completing this positive feedback loop. Next, this review focuses on the model involving embryonic exposure to these rewarding substances. In addition to revealing a similar positive feedback circuit, this model greatly advances our understanding of the diverse changes that occur in these neuropeptide/molecular systems in the embryo and how they relate, perhaps causally, to the disturbances in behavior early in life that predict a later increased risk of developing substance use disorders. Studies using this model demonstrate in animals that embryonic exposure to these rewarding substances, in addition to stimulating the expression of peptide neurons, increases the intracellular molecular systems in neuroprogenitor cells that promote their development. It also alters the morphology, migration, location and neurochemical profile of the peptide neurons and causes them to develop aberrant neuronal projections to forebrain structures. Moreover, it produces disturbances in behavior at a young age, which are sex-dependent and occur in females more than in males, that can be directly linked to the neuropeptide/molecular changes in the embryo and predict the development of behavioral disorders later in life. These results supporting the close relationship between the brain and behavior are consistent with clinical studies, showing females to be more vulnerable than males to developing substance use disorders with co-occurring emotional conditions and female offspring to respond more adversely than male offspring to prenatal exposure to rewarding substances. It is concluded that the continued consumption of or exposure to rewarding substances at any stage of life can, through such peptide brain systems, significantly increase an individual’s vulnerability to developing neurological disorders such as substance use disorders, anxiety, depression, or cognitive impairments.

## 1. Introduction

Extensive research in humans and animals supports the involvement of multiple brain systems in mediating reward-motivated and emotional behavior related to the consumption of rewarding substances including commonly used drugs like alcohol, nicotine and cocaine, as well as palatable fat-rich food, and it provides some evidence that repeated exposure to these substances can in turn lead to their overconsumption and abuse along with emotional disturbances. To understand the role of these brain systems in mediating this positive feedback loop, this review focuses on the neuropeptide melanin concentrating hormone (MCH). This peptide system is expressed in a localized neuronal population of the hypothalamus, stimulates the dopamine (DA) system that mediates reward and has dense projections to limbic and striatal forebrain regions that are shown to densely express MCH receptors and have an important role in mediating reward-motivated and related emotional behavior.

**Figure 1:**
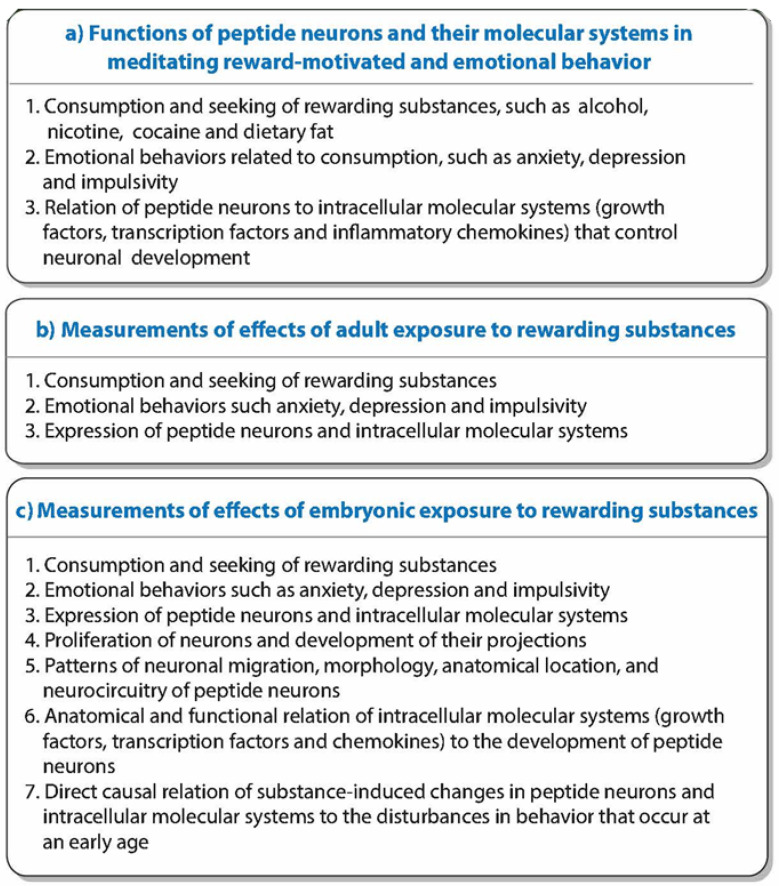
This graphical abstract summarizes: (**a**) functions of peptide neurons; (**b**) measurements of effects of adult exposure to rewarding drugs and fat-rich food; and (**c**) more in-depth measurements of effects of embryonic exposure to these substances.

As outlined in Figure 1, this review first summarizes evidence supporting this function of the MCH neurons, and it also describes the functions of the closely related hypocretin/orexin (HCRT) peptide neurons and the molecular mechanisms within these peptide neurons that promote their development. It then describes how continued consumption of or exposure to the rewarding substances in adults and adolescents further stimulates these neuropeptide and molecular systems, leading to the abuse of these substances and development of neurological conditions like substance use disorders (SUDs) with co-occurring emotional conditions. To better understand this complex process and the sequence of events involved, this review next focuses on the model involving prenatal exposure to the rewarding substances in rodents or direct embryonic exposure in zebrafish. These animal models allow a more in-depth examination of the effects produced by these rewarding substances, both on the peptide neurons and their molecular systems in the embryo and on the reward-motivated and emotional behavior that occurs at a young age. It also enables one to test more directly whether these changes in the peptide neurons and molecular systems do, in fact, contribute to the behavioral disturbances and perhaps sex differences that may develop early in life long before puberty.

## 2. Role of MCH Neurons and Related Neuropeptide and Molecular Systems in Mediating Reward-Motivated Behavior

**Figure 2:**
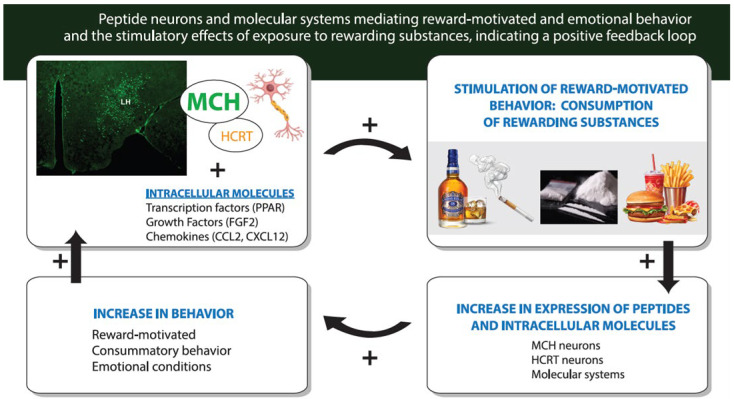
Diagram illustrating the role of hypothalamic MCH neurons (shown in green photomicrograph) along with closely related HCRT neurons and their intracellular molecular systems in mediating reward-motivated behavior, and also the positive feedback loop that exists between the consumption of rewarding substances such as alcohol, nicotine, cocaine and a palatable fat-rich diet and the stimulation of these peptide neurons and the molecular systems such as transcription factors, growth factors and inflammatory chemokines that lead to a further increase in the behavior. Abbreviations: MCH, melanin-concentrating hormone; HCRT, hypocretin/orexin peptide; PPAR, peroxisome proliferator-activated receptor; FGF2, fibroblast growth factor 2; CCL2 and CXCL12, inflammatory chemokines.

The literature summarized here as shown in Figure 2 provides support for the idea that MCH neurons concentrated in the lateral hypothalamus (LH) and positively related to DA reward have an important role in stimulating reward-motivated and emotional behavior including the consumption of rewarding drugs and palatable food. These MCH neurons are anatomically and functionally linked to neurons expressing HCRT, and both of these peptide neurons have intracellular molecular systems that promote their development and ultimately their function. The evidence also illustrated in Figure 2 and described in the next section shows how these MCH neurons, along with the HCRT neurons and their intracellular mechanisms, are further stimulated by the consumption of or exposure in adults or adolescents to these rewarding substances that leads to a greater increase in consummatory and emotional behavior, reflecting a positive feedback loop.

### 2.1. Function of MCH Neurons in the LH in the Control of Reward-Motivated and Related Emotional Behavior

There is a large body of evidence in animal studies that positively links MCH neurons in the LH to the consumption of rewarding substances, including commonly used drugs and palatable food, which stimulate the mesolimbic DA system that mediates their reinforcing properties [1,2,3]. The co-expression of MCHR1 with DA receptors in the nucleus accumbens [4] suggests that MCH through its receptor interacts directly with the DA system, increasing both the release of DA in the nucleus accumbens [4] and stimulating the intake and rewarding properties of commonly abused drugs as well as palatable food [4,5,6]. Studies demonstrate that the central administration of MCH strongly increases food intake [7,8,9], the overexpression of endogenous MCH leads to overeating [10] and the administration of MCHR1 antagonists or knockout of the MCH gene significantly reduces food intake [11,12,13]. This stimulatory effect of MCH on feeding behavior is the strongest with palatable fat-rich diets, indicating the importance of their rewarding properties. This is shown by evidence that MCH activity and chronic MCH treatment or overexpression increase the preference for highly palatable food and the susceptibility toward greater high-fat feeding and obesity [14,15], and a deficiency of MCH or antagonism of the MCHR1 receptor reduces the motivation to consume a fat-rich diet [14,16]. The MCH system is also linked to the consumption of rewarding drugs, including alcohol, nicotine and cocaine. A relation of MCH to alcohol intake is demonstrated by evidence that endogenous expression of MCH is positively correlated with the intake of alcohol [17], and the injection of MCH into the hypothalamus or nucleus accumbens stimulates alcohol drinking and operant responses for alcohol while having no effect on the ingestion of water or dry food [17,18,19]. Also, blockade of the MCH system causes a decrease in alcohol consumption, with peripheral administration of an MCHR1 antagonist reducing alcohol self-administration and cue-induced reinstatement of alcohol-seeking [20], and treatment with an MCHR1 antagonist or genetic deletion of MCHR1 decreasing alcohol-induced conditioned place preference [21]. Studies of nicotine yield similar results showing that peripheral administration of an MCH receptor antagonist reduces nicotine-induced symptoms including locomotor behavior [22]. Further, investigations of cocaine demonstrate that the level of MCH neuronal activity positively predicts cocaine-seeking, knockout of the MCHR1 gene attenuates cocaine responses and reduces preference for a cocaine-paired chamber, and acute blockade of the MCH system suppresses cocaine self-administration and cocaine-induced reinstatement [4,23].

In addition to consummatory behavior, studies in rodents positively link the MCH system to emotional behavior that accompanies reward-driven behavior. This is demonstrated by evidence that the microinjection of MCH and chemogenetic activation of MCH neurons induces anxiety [24,25], and central injection of MCH causes depressive-like behavior [26,27]. In addition, the administration of MCH receptor antagonists is shown to have both anxiolytic and antidepressant effects [24,28,29]. Further, there is evidence that impulsive behavior is produced by a site-specific pharmacological or chemogenetic upregulation of MCH communication to the hippocampus [30].

### 2.2. Function of HCRT Peptide Neurons Closely Related to MCH Neurons in the Control of Reward-Motivated Behavior

There are other neuropeptide systems in the LH that are anatomically close to the MCH neurons and functionally similar in promoting reward-motivated and emotional behavior. One in particular is HCRT, which like MCH is expressed only in hypothalamic neurons while having projections throughout the brain and has an important role in mediating the rewarding properties of drugs and palatable food and the motivation to seek and consume these substances, with high expression of the HCRT reserve pool contributing to hypermotivation for drugs and addiction [31,32,33,34]. As with MCH, the HCRT neurons are concentrated in the LH, and while MCH and HCRT mostly exist in separate populations [35], these two peptides exhibit some colocalization, with a three-dimensional culture of mouse embryonic stem cells showing 10% of the MCH neurons to be immunoreactive for HCRT [36]. Also, these two populations of peptide neurons are reciprocally interactive, with MCH neurons shown to co-express HCRT receptors [37]; exhibit multiple contacts with HCRT neurons along their soma, dendrites and axons with reciprocal synaptic relationships [35,38]; and receive inhibitory signaling via local microcircuits from HCRT neurons [39]. Further, while having some opposing roles in sleep/wake and fasting/feeding cycles and metabolic sensing [40,41], these two populations of peptide neurons are similar in exhibiting a positive relationship to DA and rewarding drugs and palatable food and stimulating the seeking and consumption of these substances. Like MCH, HCRT stimulates the release of DA [42,43]; mediates the reinforcement of drug-seeking behavior for all major drug classes including alcohol [44,45], nicotine [46,47] and cocaine [48,49]; and is positively associated with the development of emotional disorders such as anxiety and depression [33,50]. A direct functional interaction between the MCH and HCRT systems in their role in promoting operant responses for drug and food rewards is demonstrated at the molecular level, with a chronic knockdown of HCRT causing a reduction in the number of MCH neurons and this decrease in both peptides associated with a reduced motivation to consume cocaine and a palatable diet along with no effect on water or dry food intake [51].

Along with HCRT neurons is the peptide, dynorphin (DYN), which is shown to colocalize in most HCRT neurons [52] and is co-expressed with MCHR1 [6]. While anatomically close within the hypothalamus, this opioid peptide is expressed throughout multiple brain areas and differs markedly from MCH and HCRT in its function. It has a negative relationship to mesolimbic DA neurotransmission and reward-motivated behavior, serving through presynaptic κ-opioid receptors as a negative feedback mechanism to inhibit DA release in the nucleus accumbens shell [53,54]. DYN also blocks many neurochemical and behavioral responses evoked by rewarding substances [53,54,55,56,57,58], exerts aversive and anxiogenic effects that influence consummatory behavior [55,56,59] and causes a dysregulation that is linked to drug addiction and overeating of palatable food and interacts with HCRT to regulate drug-seeking and self-administration behaviors [60,61]. There is another peptide, cocaine- and amphetamine-regulated transcript (CART), which is expressed throughout the brain and is detected in some MCH neurons that project to forebrain areas involved in mediating reward-driven behavior [36,62,63] and some MCH terminals in the ventral tegmental area that make contact with DA neurons [64,65]. Like MCH and HCRT, this peptide stimulates the release of DA [66,67], and it is positively related to consummatory behavior [63]. Although there is evidence that CART may inhibit the re-instatement of drug-seeking behavior, its injection directly into specific hypothalamic nuclei including the LH stimulates food intake; its overexpression in the hypothalamus is positively related to an increase in food intake; and its gene knockout causes a reduction in alcohol consumption [63,68]. The endogenous expression of CART is also positively related to certain emotional behavior associated with substance abuse, including anxiety and depression as shown in rodents [69,70] and depressive behavior exhibited in suicide victims [71].

### 2.3. Function of Intracellular Molecular Systems That Control Development of Peptide Neuron and Reward-Motivated Behavior

Despite the many studies describing the function of MCH and related peptide systems like HCRT in the control of reward-motivated behavior, the molecular systems including transcription factors, growth factors and inflammatory chemokines indicated in Figure 2 that may exist within these peptide neurons and are involved in stimulating their development, migration and ultimately their function remain poorly understood. There is a recent in vitro study of MCH neurons which demonstrates the importance of Hedgehog signaling in producing neurochemical subtypes of MCH neurons [36], with the absence of exogenous Hedgehog signals promoting the differentiation of MCH neurons that co-express CART and the presence of Hedgehog signaling causing some MCH neurons to lack the CART peptide. There is other evidence suggesting a role of the transcription factor peroxisome proliferator-activated receptor (PPAR) in the development of MCH neurons, with one of its three isoforms PPARβ/δ shown to be at high levels in the hypothalamus [72,73] and densely expressed within the MCH as well as HCRT neurons in the LH [73]. With PPARs known to have an important role in promoting the proliferation, differentiation and maturation of neurons [74,75,76], this colocalization of PPARβ/δ with these peptide neurons suggests its potential role in stimulating their development. The possibility that PPARs also have a function in promoting drug use and palatable food intake is supported by evidence that they are expressed in midbrain DA neurons [77,78] and modulate DA release [77,79]. Further, the peripheral administration of PPARs in rodents affects the consumption of alcohol and nicotine [79,80,81], and PPARγ administered to humans modulates craving in cocaine use disorder [82] and mediates the overeating induced by a fat-rich diet [83].

The involvement of growth factors in mediating the development and function of the peptide neurons is indicated by a study of the vascular endothelial growth factor A, which is expressed in MCH neurons and has a role in regulating the permeability of the median eminence that modulates energy homeostasis and sleep [84]. It is also suggested by another study of the fibroblast growth factor 2 (FGF2) and its receptor FGFR1 which are linked to alcohol consumption and other behaviors associated with alcohol use disorder [85]. This growth factor system is shown to be localized in neurons as well as non-neuronal cells [86,87,88], have an important function in stimulating cell proliferation and differentiation during brain development [88,89,90] and increase the activity of mesolimbic and nigrostriatal DA neurons [91]. Both FGF2 and FGFR1 are found to be heavily expressed in MCH neurons as demonstrated in newborn and adolescent rodents [92]. They are also positively related to the consumption of rewarding substances, with the drinking of alcohol shown to be stimulated by the peripheral or forebrain injection of FGF2 and reduced by FGF2 deficiency, systemic administration of an FGFR1 antagonist and striatal infusion of an anti-FGF2 neutralizing antibody [91,93,94,95]. Moreover, the acquisition of cocaine self-administration is increased by systemic recombinant FGF2 treatment [96], rodents selectively bred for a low novelty response have lower FGF2 in association with reduced addictive behaviors related to cocaine self-administration [97] and the disruption of FGF2 signaling enhances thermogenesis and protects against weight gain and the development of obesity [98].

There is further evidence relating neuroimmune pathways to rewarding drugs, with levels of inflammatory chemokines shown to predict the development of addiction to certain drugs [99,100,101,102,103] and neurological disorders associated with SUDs [104,105]. Building on early evidence showing MCH to have immunomodulatory properties in the periphery [106], studies focusing on MCH neurons in the LH examined the inflammatory chemokine systems of CCL2 and its receptor CCR2 [107,108,109,110] and CXCL12 and its receptor CXCR4 [111]. These systems are key mediators in the molecular pathways positively linking the immune system to neuronal development and function [112,113,114] and involved in stimulating the proliferation and migration of hypothalamic neurons and the development of their projections [115,116,117,118]. Both CCL2 and CCR2 are found to be heavily expressed in MCH neurons of the LH [107,108,119]. Like MCH, this chemokine system is closely related to reward pathways, with CCL2 shown to stimulate striatal DA release [120], and it is also positively linked to the overconsumption of rewarding substances like alcohol and a high-fat diet [121,122,123,124], to cocaine-induced locomotor sensitization [125] and to behaviors such as anxiety and locomotor activity [126,127,128].

While also detected in MCH neurons, CXCL12 and CXCR4 are found in rodents to be more heavily expressed in HCRT neurons [111]. In zebrafish, the homologues CXCL12a and CXCR4b are also shown to colocalize with HCRT neurons under control conditions [129,130], and the overexpression of CXCL12a in embryos increases the number of HCRT neurons and the density of their projections [117], while influencing the firing of MCH neurons [131]. Furthermore, the CXCL12/CXCR4 system stimulates the proliferation, differentiation and migration of neurons in the hypothalamus and other areas [132,133,134,135], and it has a positive relationship to the DA system [136]. The involvement of this chemokine system in the consumption of drugs and palatable food is supported by evidence that the ingestion of a palatable high-fat diet is stimulated by CXCL12 injection into the third ventricle while being reduced by a genetic CCR2 deficiency [134]. Also, the administration of a CXCR4 receptor antagonist blocks a cocaine-induced increase in conditioned place preference and locomotor activity [137], and endogenous CXCR4 is positively associated with behaviors like locomotor activity and depression that are linked to excess consummatory behavior [138].

### 2.4. Conclusions

Together, this evidence as indicated in Figure 2 demonstrates that the MCH neurons along with HCRT neurons in the hypothalamus, which stimulate DA neurotransmission and project to limbic and striatal brain areas that have an important role in reward-motivated behavior and related emotional behavior, function normally in stimulating these behaviors and have intracellular molecular systems that can mediate the embryonic development and ultimate behavioral function of these peptide neurons.

## 3. Adult Substance Exposure and Stimulation of the Peptide Neurons, Their Molecular Systems and Behavior

Building on results supporting the role of these neuropeptide and molecular systems in mediating reward-motivated and emotional behavior, this section reviews evidence showing how the consumption of or exposure in adolescents and adults to rewarding substances, including commonly used drugs and palatable food, can further stimulate these neural systems and their behavioral functions, reflecting a positive feedback loop that leads to an increase in drug use and palatable food intake and related emotional disturbances, as illustrated in Figure 2.

### 3.1. Adult Exposure to Rewarding Substances and Stimulatory Effects on Reward-Motivated and Emotional Behavior

Clinical studies show how continued consumption of or exposure to rewarding substances can have major behavioral consequences in adults, as well as during adolescence when significant structural, functional and neurochemical changes are occurring in the brain [139,140]. Chronic use of alcohol, nicotine or cocaine is associated with an increase in anxiety, impulsivity and depression, along with inattention and cognitive deficits [141,142,143,144,145] and an increased risk for developing SUDs [146,147] and other neurological disorders [148,149,150]. A similar outcome is produced by chronic consumption of a fat-rich diet, which is accompanied by greater drinking of alcohol [151,152] along with the development of depression and cognitive impairments [153]. Studies in adult and adolescent rodents also describe behavioral disturbances associated with chronic exposure to or consumption of these rewarding drugs and palatable food. Alcohol or nicotine exposure leads to an increase in hyperactivity, anxiety, impulsivity and cognitive impairments along with greater alcohol intake [110,154,155,156,157,158], and the consumption of or exposure to nicotine is suggested to serve as a gateway to the use of other drugs [159], causing increased susceptibility to the co-use of nicotine with alcohol and the self-administration of both alcohol and cocaine [160,161,162] together with an increase in anxiety-like behavior and cognitive deficits [163,164]. Moreover, chronic consumption of a fat-rich diet is associated with an increase in depression and cognitive deficits, and it can lead to elevated alcohol drinking and motivation to obtain alcohol, earlier initiation of nicotine intake and greater cocaine-seeking and -taking behavior [153,165,166,167,168,169]. Rodents initially characterized as prone to overconsuming a fat-rich diet are shown to drink more alcohol [167] and exhibit greater anxiety and locomotor activity [15].

### 3.2. Adult Exposure to Rewarding Substances and Stimulatory Effects on Endogenous Expression of MCH Neurons

Studies of the brain show these behavioral disturbances in adults or adolescents induced by chronic exposure to drugs or fat-rich food to be accompanied by a strong and consistent stimulatory effect on endogenous MCH expression in the LH, further supporting this peptide’s role in mediating the positive feedback loop between these rewarding substances and reward-motivated behavior. This increase in MCH is demonstrated with alcohol, which after acute administration but not prolonged intake increases the gene expression of MCH neurons in an anatomically specific way, in the LH but not the nearby zona incerta [18]. It is similarly observed with cannabinoids which increase MCH neuronal activity in hypothalamic slices in vitro [170] and with cocaine which alters the expression profile of MCH neurons and their electrophysiological properties [171]. The consumption of a high-fat diet also increases excitatory neurotransmission in MCH neurons [15,172], and animals prone to overconsuming this palatable food have a markedly higher expression of MCH neurons [15].

### 3.3. Adult Exposure to Rewarding Substances and Stimulatory Effects on HCRT Neurons Closely Related to MCH Neurons

Similar to their impact on MCH neurons, the consumption of rewarding drugs and palatable food in adults and adolescents has a stimulatory effect on HCRT neurons in the LH, which interact closely with MCH neurons and are positively related to DA and reward. Acute or chronic exposure in rodents to alcohol or nicotine increases the expression of HCRT neurons in the LH together with MCH [173], and a similar stimulatory effect on HCRT is produced by acute and chronic consumption of a palatable high-fat diet [15,174,175,176]. While the expression of CART in the LH that is positively related to reward is similarly stimulated by the acute administration or seeking of alcohol [177,178], the effects of rewarding substances on DYN which colocalizes with HCRT but is negatively related to DA reward are inconsistent, as shown by measurements in different extra-hypothalamic regions [179,180,181] and in the hypothalamus where DYN expression is reduced by nicotine exposure [182] while being elevated in alcohol-preferring rodents [183]. Although exposure to stimulants or opioids stimulates HCRT neurons, it seems to have little effect on MCH neurons [184,185], a difference between these two peptides that needs to be explained by further studies.

### 3.4. Adult Exposure and Effects on Intracellular Molecular Systems That Control Development and Function of Peptide Neurons

Molecular systems such as transcription factors, growth factors and chemokines, which are found to be expressed in both MCH and HCRT peptide neurons and are likely involved in mediating their development and behavioral functions, are also shown like the peptides to be stimulated in the brain or periphery by exposure in adults to rewarding drugs and palatable food. The administration of alcohol in adult rodents also stimulates the expression of PPARγ in the brain as well as its periphery [186,187,188], and cocaine exposure activates PPARγ in the striatum [189]. Expression of the growth factor FGF2 is similarly increased in the prefrontal cortex by cocaine use in human subjects [190], and FGF2 and its receptor FGFR1 are both stimulated in the brain of adult rodents by the consumption of alcohol, nicotine, cocaine and a fat-rich diet [93,191,192,193,194]. Studies of the chemokine systems in adults reveal similar stimulatory effects of rewarding substances on CCL2 and its receptor CCR2. In clinical reports, the excessive use of alcohol increases chemokines in the brain and periphery to levels positively related to the craving for alcohol [99,100,101], and alcoholics have significantly elevated CCL2 levels in the blood, peripheral organs and cerebrospinal fluid [195,196,197], as well as in limbic brain regions [99]. In adult rodents, acute and chronic administration of alcohol also stimulates CCL2 mRNA and protein levels in different brain areas and the periphery, an effect that persists for days [198,199], and chronic alcohol administration stimulates the expression of CCL2 in the hippocampus [200] and CCR2 in peripheral tissue [201]. Further, the administration of nicotine is shown to increase the levels of various chemokines including CCL2 in microvessels of the brain [202]; cocaine administration stimulates CCL2 secretion in brain endothelial cells [203], CCR2 expression in monocytes in vitro [204] and CCR5 mRNA in the mesolimbic system [205]; and chronic consumption of a fat-rich diet stimulates the expression of CCL2 and CCR2 in the intestines [206]. Studies of CXCL12 and its receptor CXCR4 in rodents show this system to be similarly stimulated by rewarding substances. For example, alcohol exposure increases CXCL12 in the periphery [207], the consumption of fat-rich diet stimulates CXCL12 levels in circulating immune cells [208,209] and the expression of CXCL12 and CXCR4 in the LH and other hypothalamic nuclei [134], and cocaine exposure increases CXCL12 gene expression in the midbrain and plasma [102,137].

### 3.5. Conclusions

These studies support a positive feedback loop between the rewarding substances and reward-motivated behavior which is mediated by MCH and closely associated HCRT neurons in the hypothalamus that are positively related to DA and reward. As illustrated in Figure 2, the results demonstrate that further exposure in adults and adolescents to these rewarding substances has strong and consistent stimulatory effects on the peptide neurons and the molecular systems that promote their development, likely contributing to the overconsumption and abuse of these substances and the development of neurological disorders such as SUDs.

## 4. Prenatal Substance Exposure and Effects on Embryonic Development of MCH Neurons in Rodent Offspring

**Figure 3:**
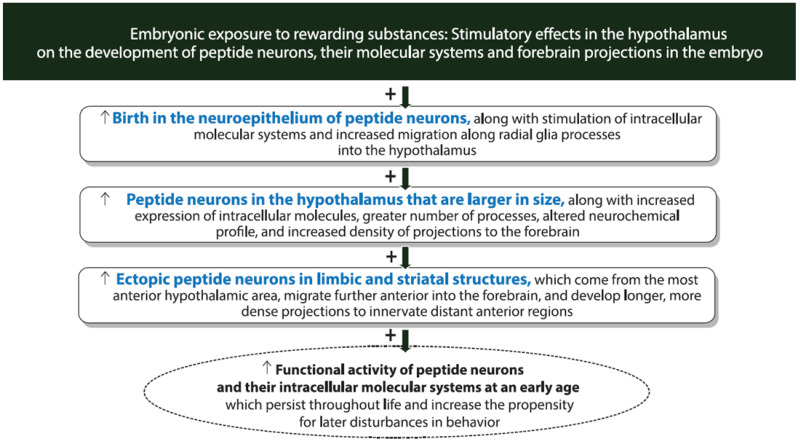
Diagram showing diverse effects of embryonic exposure to rewarding substances on the development of hypothalamic neuropeptide and molecular systems. In addition to stimulating the expression of MCH and HCRT neurons in the hypothalamus, embryonic exposure to rewarding substances at relatively low concentrations strongly stimulates the birth, development, migration and location of the peptide neurons along with their intracellular molecules and affects their morphology, processes and projections to distant forebrain regions that may promote changes in reward-motivated behavior throughout life that increase the risk of developing substance-use disorders.

To further investigate this positive feedback loop involving the peptide neurons and intracellular molecules as revealed by exposure in adults or adolescents to rewarding substances (Figure 2), this section focuses on the model involving prenatal exposure to rewarding drugs and palatable food. This model, which in animals generally tests these substances at relatively low concentrations and with short periods of exposure, allows one to examine the neuronal and molecular systems as they develop early in the embryo in response to the rewarding substances. Like the effects of adolescent and adult exposure, these studies show that prenatal exposure to rewarding substances has a consistent stimulatory effect on the expression of MCH neurons in the offspring. With more in-depth analyses of these neuronal changes in the embryo, further evidence summarized in Figure 3 demonstrates that prenatal exposure to the different rewarding substances has a range of effects in the embryo, on the birth, migration, morphology and processes of the MCH neurons, leading to their increased expression in the LH and causing some MCH neurons to migrate into more anterior structures where they are not normally found.

### 4.1. Prenatal Exposure to Rewarding Substances and Effects on MCH Neurons in Postnatal, Adolescent and Adult Offspring

Prenatal exposure to rewarding substances in rodents is shown to stimulate the expression and density of MCH neurons in the LH of the offspring at different ages, during the postnatal period as well as in adolescent and adult offspring [92,109,111,210,211]. This stimulatory effect of prenatal exposure on MCH expression is found to be time- and dose-related, observed when alcohol is presented for a short period from embryonic days 10–15 when MCH neurons are born, migrate and mature [212,213,214,215] and at relatively low doses that increase blood alcohol levels to <150 g/dL but not at high doses that increase blood alcohol to higher levels which disrupt the development of neurons while stimulating glial cells [92,108,109,110]. This stimulatory effect on MCH neurons is anatomically localized, seen in the dorsal region of the LH but not in the ventral LH region nor in the basal area of the hypothalamus [92], and it occurs in the absence of changes in glial cells, as shown with measurements of astrocytes, oligodendrocytes or microglia [210]. Further, it is similarly seen after prenatal exposure to nicotine and after prenatal exposure to a fat-rich diet [210,211], indicating that MCH neurons in the embryo are responsive to a broad range of rewarding substances including commonly used drugs and palatable food.

### 4.2. Prenatal Exposure to Rewarding Substances and Effects on Development and Morphology of MCH Neurons in the Embryo

Examination of the embryo before birth yields further information about the substance-induced changes that occur early in the development of the MCH neurons in rodents. Prenatal exposure to alcohol at low doses increases the proliferation and differentiation of MCH neurons in the neuroepithelium around the third ventricle [111,216], where neurons destined for the LH are born [217]. Similar effects on developing MCH neurons are seen with prenatal exposure to nicotine at a low dose, which stimulates neurogenesis but not gliogenesis and increases the number of newly generated MCH neurons [211]. They are also produced by prenatal exposure to a fat-rich diet for a short period. This increases cells in the neuroepithelium that differentiate specifically into neuronal restricted precursors or immature neurons but again causes no change in glial-restricted precursors, glia or tanycytes in the neuroepithelium, underscoring a particular sensitivity of these neurons to the rewarding substances during their embryonic development [210].

In addition to stimulating neuronal precursor cells, prenatal exposure to alcohol alters the morphological characteristics of MCH neurons in the LH as shown in postnatal offspring. Prenatal alcohol exposure in rodents causes them to become larger in size than control neurons, and it leads them to develop a greater number of processes emanating from the soma [218]. These changes in neuronal morphology are consistent with results from studies examining other neuronal systems at a young age. They show that prenatal nicotine exposure alters the dendritic branching and spine density of neurons in the nucleus accumbens and medial prefrontal cortex [219,220]. Further, prenatal cocaine exposure affects the dendritic outgrowth of cortical neurons [221,222], and prenatal high-fat diet exposure changes the morphology and number of dendrites and spines of neurons in the amygdala and hippocampus [223,224].

### 4.3. Prenatal Exposure to Rewarding Substances and Effects on the Migration and Location of MCH Neurons in the Embryo

The migratory pattern and location of MCH neurons in the embryo are also altered by exposure to rewarding drugs and palatable food as shown in rodents. Examination of the hypothalamic neuroepithelium, where neurons are born and radial glia progenitor cells project laterally to provide scaffolds for neuronal migration into the LH [225,226], demonstrates that prenatal alcohol exposure at a low dose increases the density and processes of radial glia cells but not microglia and the number of MCH neurons in close proximity to the radial glia cells and making contact along their processes projecting into the LH [216]. By promoting premature neuronal migration or extending it, alcohol also alters the ultimate location of the MCH neurons in the embryo brain, causing them to become ectopically expressed outside of the hypothalamus [218]. These ectopic MCH neurons are detected in more anterior brain regions, specifically the nucleus accumbens and caudate putamen where these neurons have not previously been observed, and they are still evident in postnatal stages as well as adolescent and adult offspring, indicating that this effect of alcohol is long-lasting [218]. These ectopic neurons, while smaller in size, are mature with processes, and they are likely to be active and integrated into the local neurocircuitry and may even be more excitable, as suggested by evidence that a smaller surface area can produce action potentials with a lower input [227]. These results from analyses of MCH neurons in the embryo are consistent with investigations of other neuronal systems involving prenatal exposure to different rewarding substances. These reports show that alcohol causes heterotopias of cortical neurons [228] as shown in the brains of children with fetal alcohol spectrum disorder [229], nicotine increases the rate of neuronal migration in the hippocampus [230], cocaine causes hippocampal pyramidal cells to be ectopically expressed in the striatum [231] and a fat-rich diet stimulates the migration of postmitotic neurons in the hypothalamus [210].

### 4.4. Conclusions

These findings in rodents as summarized in Figure 3 demonstrate that prenatal exposure at low levels to rewarding drugs or palatable food, in addition to increasing the expression of MCH neurons in the LH of the offspring as shown with adult and adolescent exposure, has diverse effects on the early development of these neurons as demonstrated in the embryo. These effects include an increase in their birth and differentiation in the hypothalamic neuroepithelium, changes in their morphology and processes and alterations in their migration leading to their ectopic expression in brain structures further anterior to the hypothalamus.

## 5. Embryonic Substance Exposure and the Effects on HCRT Neurons in the Hypothalamus of Rodents and Zebrafish

With evidence showing HCRT neurons to be anatomically and functionally linked to MCH neurons, the research described here examines in rodents whether these HCRT neurons are similar to MCH in their responses to prenatal exposure to the rewarding substances, as suggested by the effects described above with adult and adolescent exposure. It also examines in zebrafish whether the HCRT neurons can be stimulated by direct exposure of the embryo to a low dose of alcohol placed in the water. The results of these studies in rodents and zebrafish as summarized in Figure 3 demonstrate that, while stimulating embryonic development of HCRT neurons with changes in their birth, morphology, migration and location as shown with MCH neurons, these rewarding substances alter the neurochemical profile of the HCRT neurons and stimulate their projections, causing them to innervate new and more distant forebrain regions.

### 5.1. Prenatal Exposure to Rewarding Substances and Stimulatory Effects on HCRT Like MCH Neurons in Rodent Offspring

As demonstrated with adult exposure, prenatal exposure to drugs or a palatable food in rodents stimulates the HCRT peptide system in the offspring. As with MCH, the expression of HCRT neurons in the LH is significantly increased by prenatal exposure to alcohol, which also stimulates their proliferation in the embryo and increases their size and number of processes from the soma [108,111,218]. In addition, alcohol exposure causes some HCRT neurons to be ectopically expressed, further anterior in the nucleus accumbens and caudate putamen where they have not previously been detected [218]. These stimulatory effects of alcohol on HCRT neurons in the LH are similarly produced by prenatal exposure to nicotine [211,232] which increases their innervation of the ventral tegmental area where DA neurons are expressed [232] and by prenatal exposure to a palatable fat-rich food [210]. The expression of the CART peptide, also positively related to DA reward, is similarly simulated in the LH and ventral tegmental area by prenatal alcohol exposure [233] and by prenatal exposure to a palatable fat-rich diet [234].

### 5.2. Embryonic Exposure to Rewarding Substances and Stimulatory Effects on the Development of HCRT Neurons in Zebrafish

Further information about these stimulatory effects on HCRT neurons is provided by studies in zebrafish, an animal model that allows a more thorough examination of the brain using live imaging and 3-dimensional analyses of entire populations and is especially useful for investigating the neuronal changes induced by alcohol and other drugs [235,236]. Exposure to alcohol of the zebrafish embryo, as well as the egg before fertilization, has a stimulatory effect on the number of HCRT neurons in the hypothalamus, which as shown in rodents persists into adulthood [237,238], and it also alters the migratory path of these HCRT neurons, causing some to become ectopically expressed in areas anterior to the hypothalamus [129,218,238]. Studies of the entire population of hypothalamic neurons in zebrafish demonstrate that the effect of this rewarding substance on HCRT neurons is not seen for all neurons throughout this structure, suggesting that these peptide neurons are particularly sensitive to substance-induced stimulatory effects on their proliferation [237,238]. Moreover, a report examining the entire population of actively proliferating cells in addition to the total population of HCRT neurons reveals further insight into the nature of alcohol’s effect on these peptide neurons [239]. This study demonstrates that the most anterior region of the hypothalamus has the densest population of naturally actively proliferating cells, and it is in this specific region where embryonic exposure to alcohol has its greatest stimulatory effect on the number of HCRT neurons and causes these neurons to migrate further and become ectopically expressed in more anterior regions outside the hypothalamus.

### 5.3. Embryonic Exposure to Rewarding Substances and Stimulatory Effects on the Projections of HCRT Neurons in Zebrafish

In addition to this analysis of the entire population of HCRT neurons, the zebrafish model allows one to examine the projections of these peptide neurons and their specific neurochemical profile after embryonic alcohol exposure. In zebrafish embryos, the most anterior HCRT neurons that are most likely to become ectopically expressed outside the hypothalamus are found to have projections innervating the more anterior regions of the forebrain [239]. While having little effect on their short projections within or close to the hypothalamus, alcohol exposure has a strong stimulatory effect on their long ascending projections that terminate in the subpallium, where the density of their branch points and terminal points is increased. It additionally increases the length of these projections of the ectopic neurons, causing them to have branch points and terminal points further dorsally in the dorsal pallium where they do not normally project. These results are consistent with other findings in zebrafish showing alcohol to induce ectopic expression of oxytocin [240] and facial branchial motor neurons [241], as well as in rodents showing prenatal alcohol exposure to cause premature maturation of the HCRT neurocircuitry, increasing the branch and terminal points of their long projections to distant brain areas [242].

Analyses of HCRT, which is positively related to DA, together with the opioid peptide DYN that normally colocalizes in most HCRT neurons but is negatively related to DA reward demonstrate that embryonic exposure to alcohol differentially affects these co-expressing peptides, similar to the effect produced by adult exposure. While HCRT neurons are consistently stimulated by alcohol as well as the other rewarding substances, DYN exhibits inconsistent responses after alcohol exposure as shown across different brain regions [243,244] and shows no change in the zebrafish hypothalamus after embryonic alcohol exposure [239], consistent with the opposing roles of these peptides in mediating reward. Further analyses of the co-expression of these peptides in the same neuron demonstrate that, while alcohol has no effect on the total number of DYN transcripts or the number of HCRT neurons co-expressing DYN, it alters the neurochemical profile of the specific subpopulation of anterior alcohol-stimulated neurons that project to the forebrain, showing them to exhibit a high concentration of HCRT but no co-expression of DYN. With further studies showing DYN to have a suppressive effect both in vivo and in vitro on neurogenesis and the differentiation of neural stem cells [245,246], the absence of DYN in these stimulated ectopic HCRT neurons likely creates a more permissive environment for stimulating neurogenesis after embryonic exposure to alcohol.

### 5.4. Conclusions

As with exposure in adults and adolescents, prenatal exposure to rewarding drugs and palatable fat-rich food in rodent offspring and embryonic exposure to alcohol in zebrafish as illustrated in Figure 3 are similarly found to stimulate the development of HCRT neurons, leading to their ectopic expression in additional forebrain structures as demonstrated with MCH neurons. Further effects are revealed in zebrafish, with embryonic alcohol exposure being shown to alter the neurochemical profile of the HCRT neurons, producing high levels of HCRT with no co-expression of DYN, and increase the length and density of their projections, causing them to innervate additional and more distant forebrain areas involved in reward-motivated and emotional behavior.

## 6. Embryonic Substance Exposure and Effects on the Molecular Systems in Peptide Neurons of Rodents and Zebrafish

With embryonic exposure to rewarding substances in rodents and zebrafish being shown to affect the development and migration of MCH as well as HCRT neurons in the embryo, this section focuses on studies of the intracellular molecular systems within these peptide neurons, including the transcription factor PPARβ/δ, growth factor FGF2 and the CCL2/CCR2 and CXCL12/CXCR4 chemokine systems, which are known to stimulate neurogenesis and neuronal migration and ultimately affect behavior. The evidence described here and summarized in Figure 3, showing that the rewarding substances stimulate these molecular systems in the peptide neurons, suggests that these intracellular molecules have a role in mediating the substance-induced increase in the development of MCH and HCRT neurons in the hypothalamus.

### 6.1. Prenatal Exposure to Rewarding Substances and Effects on Transcription Factors in MCH Neurons of Rodent Offspring

While there are numerous studies of transcription factors and transduction signaling molecules showing their function in the organization of the hypothalamus and the development and connectivity of its different cellular components [247], there are only a few that have examined their role in stimulating the development of specific peptide neurons and mediating the effects of prenatal exposure to rewarding substances on neuronal development and behavior in the offspring. One study of the transcription enhancer factor-1 and its co-activator Yes-associated protein demonstrates their involvement in the stimulatory effect of adult dietary fat exposure on the peptide enkephalin [248]. Also, investigations of Sonic hedgehog signaling, showing it to be involved in the development of MCH neurons that co-express CART [36] and downregulated by prenatal alcohol exposure [249], suggest that this molecule mediates the alcohol-induced changes in the neurochemical profile of MCH neurons. There are several studies supporting a role of the PPAR transcription factor in the embryonic development of MCH neurons. They show that the expression of PPARγ is stimulated in the periphery by prenatal exposure to nicotine while being reduced in the hippocampus by prenatal alcohol exposure [250], and protein levels of PPARγ in embryonic hypothalamic neurons are increased in vitro by exposure to fatty acids [251]. Investigations of this transcription factor in relation to MCH neurons demonstrate that prenatal exposure to a palatable fat-rich diet increases the proliferation and density of PPARβ/δ-expressing neurons in the LH while stimulating the birth of MCH neurons in the offspring. It also increases the number of newly generated MCH neurons clustered in the LH that co-express high levels of PPARβ/δ [73], supporting its function in the development of these peptide neurons.

### 6.2. Prenatal Exposure to Rewarding Substances and Effects on Growth Factors in MCH Neurons of Rodent Offspring

Studies of growth factors involving prenatal exposure to rewarding substances suggest a role of FGF2 and its receptor FGFR1 in mediating their stimulatory effects on the development and function of the hypothalamic peptide neurons. Consistent with a clinical study showing that children exposed in utero to alcohol have significantly higher levels of FGF2 in their circulation [252], prenatal alcohol exposure in rodents strongly increases in postnatal offspring the expression of FGF2 and FGFR1 in the LH and the density of FGF2 and FGFR1 transcripts within the MCH neurons [92], and neonatal exposure to nicotine stimulates FGF2 expression in the hippocampus [253]. With this FGF2/FGFR1 molecular system well-known for its role in promoting neuronal development [88,89,90], its stimulation by prenatal alcohol exposure likely contributes to the increased hypothalamic neurogenesis and density of MCH neurons produced in the embryo by this rewarding substance. This is suggested by the finding that the alcohol-induced increase in FGF2 and FGFR1 transcripts in the LH is dose-dependent, occurring at a relatively low dose but not at high doses [92], similar to the dose-dependent stimulatory effect of alcohol on the density of MCH neurons [92]. It receives direct support from a report [92] showing that maternal administration of FGF2 itself on embryonic day 14 of pregnancy stimulates the birth and development of MCH neurons, similar to the effects produced by prenatal exposure to alcohol [92,108,254].

### 6.3. Prenatal Exposure to Rewarding Substances and Effects on the Chemokine System in MCH Neurons of Rodent Offspring

There are several studies suggesting that the CCL2/CCR2 chemokine system is also involved in the stimulatory effects of prenatal drug and diet exposure on hypothalamic neurogenesis and the migration specifically of MCH neurons. An in vitro study shows the expression of CCR2 to be markedly elevated in primary dissociated hypothalamic neurons from embryos prenatally exposed to a high-fat diet [255]. Similarly, in vivo studies of rodent offspring demonstrate that prenatal exposure to alcohol at low concentrations stimulates the expression in the LH of CCL2 and CCR2 specifically in neurons [108,110] while at higher doses disrupting the development of neurons and stimulating this chemokine system in glial cells [216,256], and it also increases the proliferation and density of MCH neurons that co-express CCL2 and CCR2 [108,110]. A direct role of this chemokine system in promoting the development of MCH neurons in the LH is supported by evidence that these alcohol-induced stimulatory effects in postnatal offspring are mimicked by maternal administration of CCL2 [110] and are blocked by maternal administration of a CCR2 antagonist [108,110]. This is consistent with studies in the periphery, showing that the alcohol-induced effects on neurons are suppressed by a CCR2 antagonist [201,257] and the inflammation and damaging effects on spinal cord neurons are reduced by the deletion of CCL2 [258].

Investigations of the embryo after prenatal exposure to alcohol at low doses reveal a close relationship between the CCL2/CCR2 system and MCH neurons early in development. Prenatal exposure to alcohol increases in the embryo the expression and density of CCL2 and CCR2 cells in the LH and the colocalization of CCL2 with MCH in neurons of the LH, and these effects on MCH neurons are blocked by maternal administration of a CCR2 antagonist or a CCL2 antibody that neutralizes endogenous CCL2 [109]. While CCL2 stimulated in large MCH neurons likely acts intracellularly through CCR2 to stimulate these peptide neurons, prenatal alcohol exposure also increases the density of small CCL2 neurons that surround MCH neurons where CCL2 likely acts extracellularly to stimulate CCR2 on MCH neurons, and these effects on small and large CCL2 neurons are both blocked by maternal administration of a CCR2 antagonist [110]. Further examination of the embryo, specifically in the hypothalamic neuroepithelium where neurons are born and radial glia progenitor cells projecting laterally provide scaffolds for neuronal migration into LH [225], demonstrates that prenatal alcohol exposure also increases the density and processes of radial glia cells; the colocalization of CCL2 with radial glia and neurons but not microglia; and the number of MCH neurons near to the radial glia cells that make contact along their processes projecting into LH [216]. These stimulatory effects of alcohol on radial glia neuroprogenitor cells in the embryo are similarly produced by maternal administration of CCL2 itself, and they are reversed by maternal administration of a CCR2 antagonist, providing direct evidence that the CCL2/CCR2 system in the hypothalamic neuroepithelium has an important role in guiding these immature MCH neurons toward their final destination, mainly in the LH [259].

The proposed function of this chemokine system in mediating the alcohol-induced stimulation of the neurogenesis and migration of MCH neurons is further supported by a study testing the effects of CCL2 administration directly into the cerebroventricle of the embryo. When injected on embryonic day 14, CCL2 is shown to stimulate the expression of endogenous CCL2 in radial glia cells in the neuroepithelium and their processes branching into the LH on embryonic day 19 shortly before birth, and it also increases the density of CCL2 and MCH co-expressing neurons in the LH of postnatal offspring [118]. Further evidence that other rewarding substances stimulate the CCL2/CCR2 system in the embryo is provided by clinical reports, demonstrating in newborn offspring that cocaine use during pregnancy increases the levels of different chemokines in umbilical cord blood [260] and smoking during pregnancy increases circulating levels of cytokines [261]. Also, animal studies show that prenatal exposure to nicotine stimulates CCL2 in peripheral organs of the offspring [262], tobacco smoke during pregnancy increases plasma CCL2 in infant primates [263] and prenatal exposure to a fat-rich diet causes inflammation and elevates chemokines in the brain and periphery of the offspring [255,264].

### 6.4. Embryonic Exposure to Rewarding Substances and Effect on Chemokine System in HCRT Neurons of Rodents and Zebrafish

The CXCL12/CXCR4 chemokine system is also found to be involved in the embryonic development of the hypothalamic peptide neurons. Studies in rodents demonstrate that prenatal alcohol exposure at a low dose strongly stimulates this chemokine system in the embryo and postnatal offspring, in the LH where CXCR4 cells are particularly dense and are predominantly neurons, and it increases the colocalization of CXCR4 in radial glia neuroprogenitor cells concentrated in the embryonic neuroepithelium [111]. Prenatal exposure to a fat-rich diet also increases the expression of CXCL12 and CXCR4 in the hypothalamus and the genesis of hypothalamic peptide-expressing neurons in the offspring [135]. Similar stimulatory effects of alcohol exposure on this CXCL12/CXCR4 system are described in zebrafish, revealing a cross-species involvement of this neuroimmune system in mediating these effects of drug exposure on neuronal development. A study directly relating this chemokine system to the development of HCRT neurons [130] demonstrates that embryonic exposure to alcohol at a low dose increases the number of CXCL12 and CXCR4 transcripts in the developing hypothalamus, the internalization of CXCR4 receptors in hypothalamic cells and the number of HCRT neurons that co-express CXCL12 and CXCR4, and it shows these effects to be blocked by pretreatment with a CXCR4 antagonist, supporting the involvement of this chemokine system in the alcohol-induced stimulatory effect on the development of HCRT neurons [130].

Further analyses throughout the entire zebrafish brain demonstrate that the CXCL12 transcripts and internalized CXCR4 receptors both exhibit a natural anterior-to-posterior concentration gradient, with their highest levels in the telencephalon and lowest levels in the most posterior region of the hypothalamus [129]. While maintaining these gradients, embryonic exposure to alcohol stimulates the expression of CXCL12 in the more anterior region precisely where the ectopic HCRT neurons are detected as described above, and this effect is blocked by maternal administration of a CXCR4 antagonist [129]. Also, investigations using tools of genetic manipulation in the zebrafish embryo demonstrate that the overexpression of endogenous CXCL12 in the brain mimics the stimulatory effects of embryonic alcohol exposure on the number of normally located and ectopic HCRT neurons and on the density of their long anterior projections ascending to the forebrain, and the knockdown of endogenous CXCL12 prevents these stimulatory effects of alcohol on the anterior HCRT neurons and their projections [117]. These results provide direct support for the function of this CXCL12/CXCR4 system, acting along its natural gradients, in mediating the alcohol-induced stimulation of embryonic development of HCRT neurons, including the anterior neurons that become ectopic and have long anterior projections innervating the forebrain.

### 6.5. Conclusions

It is clear from this evidence summarized in Figure 3 that these different molecular systems expressed in the MCH and HCRT neurons in the hypothalamus, including transcription factors, growth factors and neuroimmune signals, have an important role in mediating the diverse effects that embryonic exposure to rewarding substances has on these peptide neurons as shown in rodents and zebrafish. These include a stimulation of their birth and development in the neuroepithelium, their migration to and ectopic expression further anterior beyond the hypothalamus and their neurocircuitry projecting to and innervating additional and more distant forebrain regions.

## 7. Relation of Peptide and Molecular Systems to Reward-Motivated and Emotional Behavior in Rodents and Zebrafish

**Figure 4:**
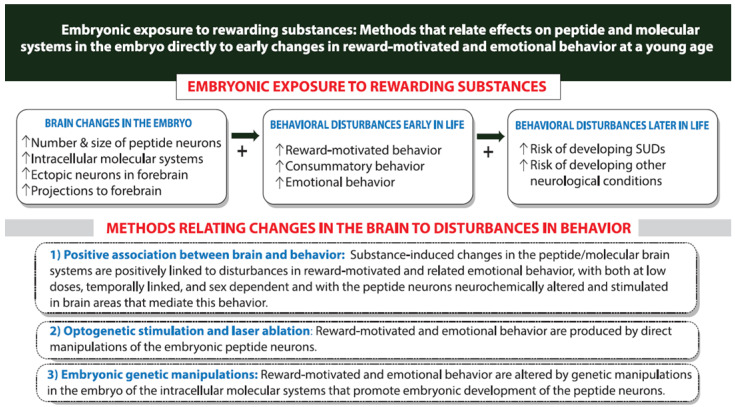
Different methods used to demonstrate a close and possibly causal relationship between the rewarding substance-induced changes in hypothalamic neuropeptide/molecular systems in the embryo and the disturbances in reward-driven and emotional behavior that occur at young age long before puberty and can strongly predict an increased risk of developing neurological conditions including substance use disorders (SUDs) later in life.

The evidence described above shows that the HCRT and MCH neurons which mediate reward-motivated behavior and their intracellular molecular systems which mediate their embryonic development are both stimulated by further consumption of or exposure to rewarding substances in adolescents and adults (Figure 2) and by exposure of the embryo to these substances (Figure 3). Building on this evidence, this section examines the possibility that the substance-induced changes in these specific neuronal and molecular systems are, in fact, involved in mediating the behavioral effects that accompany these changes in the brain. Studies of the offspring described here, using different methods as summarized in Figure 4, provide substantial support for the proposal that the stimulatory effects on MCH as well as HCRT neurons and their intracellular mechanisms are closely and possibly causally linked to the disturbances in reward-motivated and related emotional behavior that become evident at an early age and continue to develop throughout life.

### 7.1. Embryonic Exposure to Rewarding Substances and Effects on Behavior in Adult and Adolescent Offspring

Prenatal exposure to the rewarding substances in rodents like adult exposure has strong stimulatory effects in adult offspring on reward-driven behavior including the consumption of these substances and related emotional conditions, supporting the concept of a positive feedback loop between these substances and behavior. Clinical reports demonstrate that maternal consumption of alcohol during pregnancy stimulates in adult offspring the drinking of alcohol and the use of other drugs, and it increases the risk of developing neurological disorders with greater anxiety, depression, impulsivity and attention deficit hyperactivity disorder, effects that are also seen during adolescence when there is a natural surge in drug use [265,266,267,268]. These behavioral disturbances in adult offspring are similarly produced by prenatal exposure to other drugs. These include nicotine which increases the propensity for later tobacco smoking and dependence as well as for alcohol drinking [269,270,271]; cocaine which increases the likelihood of tobacco and marijuana use as well as cocaine [272,273,274]; and a palatable fat-rich diet which increases in adults the propensity to overconsume this diet and develop various neurological disorders including anxiety and depression [210,275,276].

Investigations in rodents also reveal a range of behavioral disturbances in adult and adolescent offspring after prenatal exposure to rewarding substances, even for only a few days at relatively low doses [110,277,278,279,280,281,282,283,284,285]. Prenatal exposure to alcohol is shown to produce an increase in anxiety, locomotor activity, exploration, impulsivity and alcohol-seeking, which accompany an increase in the consumption of and preference for alcohol; alcohol drinking after reinstatement during adolescence; and the vulnerability in adults of developing an addiction to alcohol and cocaine [92,108,110,278,286,287]. Also in adult offspring, prenatal exposure to nicotine at a low dose increases the self-administration of nicotine as well as alcohol and cocaine [211,288], prenatal exposure to cocaine increases cocaine self-administration [289,290] and prenatal exposure to a high-fat diet for a short period increases the preference for and drive to obtain alcohol [291] and palatable fat-rich food [292], as well as the self-administration of nicotine alone or together with alcohol [293].

### 7.2. Embryonic Exposure to Rewarding Substances and Early Effects on Behavior in Young Preadolescent Offspring

Further studies demonstrate that these behavioral effects in adult offspring produced by prenatal exposure to rewarding drugs and palatable food are apparent at an early age, long before puberty. This is described in clinical reports showing that maternal consumption of alcohol during pregnancy causes hyperactivity in preadolescent offspring as young as 9–10 years of age, and it increases their anxiety level, novelty-seeking, risk taking/exploratory behavior, impulsivity and alcohol-seeking such as increased sipping of 5% alcohol, behaviors that are predictive of later alcohol use [294,295,296,297,298,299]. Similar behavioral disturbances in preadolescent offspring are observed after prenatal exposure to other drugs. These include nicotine which increases the propensity for tobacco smoking and dependence [269,300]; cocaine which increases the likelihood of early tobacco and marijuana use and earlier initiation into the use of cocaine [272,301,302]; and a fat-rich diet which causes disordered eating at a young age including chronic overconsumption of highly palatable food [303,304].

These behavioral effects in clinical studies that develop early in life after prenatal exposure to rewarding substances and predict later behavioral disturbances are similarly observed in rodent offspring, during preadolescence or even before weaning as well as in adulthood. Prenatal exposure to alcohol causes an increase in anxiety, exploration, impulsivity and alcohol-seeking behavior in offspring as young as 12 days of age [305,306], with similar behavioral changes also produced in postnatal offspring by prenatal exposure to nicotine [307,308] and prenatal exposure to cocaine [309]. Notably, these early behavioral effects in rodents are also observed in zebrafish at a young age. Exposure of the zebrafish embryo to alcohol in the water, at a low dose and for only 2 h, causes a variety of behavioral changes in larval fish a few days later including an increase in locomotor activity, anxiety-like behavior, impulsivity, novelty-seeking, exploration and alcohol-seeking behavior [218,305]. Interestingly, these same behavioral disturbances that develop early in zebrafish are also seen after alcohol exposure of the egg itself before paternal fertilization [310].

### 7.3. Relation of Embryonic Substance-Induced Changes in MCH Neurons to Behavioral Disturbances in Young Rodents

These early behavioral changes produced in rodents by prenatal exposure to rewarding substances at low doses are consistently accompanied by a stimulatory effect on the expression and density of MCH neurons in the embryo and after birth in postnatal offspring [92,109,111,211,218]. A close and possibly causal relationship between this change in the brain and the disturbances in reward-motivated and emotional behavior observed in young offspring is further supported by investigations of the embryonic development, maturation and migration of MCH neurons. These studies demonstrate that the behavioral changes produced by prenatal alcohol exposure are seen specifically at the low doses that are also effective in stimulating MCH neurogenesis in the embryo [109,110], and they are apparent at an early age before weaning within 12 days after birth when the density of MCH neurons is elevated [305]. These reports also show that the alcohol-induced increase in expression of MCH neurons is strongly positively correlated with the different behavioral measures at a young age, including an increase in alcohol consumption and other reward-motivated or emotional drug-related behaviors [15,108,110,311,312].

Further evidence demonstrates that the stimulation of MCH neurons is anatomically localized, in a specific area known to have important functions in mediating reward-motivated behavior. This is demonstrated with acute alcohol injection, which stimulates MCH neurons in the LH—which mediates reward-driven behaviors—but suppresses those neurons in the nearby zona incerta that mediate locomotor but not reward-seeking behaviors [18,313,314,315]. It is also shown with prenatal alcohol exposure, which increases MCH neurons in the dorsal region of the LH that mediates reward-motivated behavior as indicated by studies of cocaine [316,317] but not in the ventral region that mediates other functions such as positive emotional behavior and arousal [318,319]. Moreover, the prenatal alcohol-induced ectopic MCH neurons outside of the hypothalamus, which are detected in the more anterior nucleus accumbens and caudate putamen structures that mediate alcohol-related behaviors and still evident in newborn and early adolescent offspring [218,320,321], also likely contribute to the reward-motivated behavior in the offspring. Together with the above evidence in adolescents and adults that MCH neurons mediate reward-motivated and emotional behavior and are in turn stimulated along with behavior by the rewarding substances, these studies of the embryonic brain and young offspring behavior after prenatal substance exposure support the idea that MCH neurons have an important function in mediating the behavioral disturbances that occur early postnatally. They also predict an increased vulnerability throughout life of the offspring overconsuming the rewarding substances and developing disturbances in emotional behaviors as shown in SUDs.

### 7.4. Relation of Embryonic Substance-Induced Effects on HCRT Neurons to Early Behavioral Disturbances in Young Rodents

As with MCH neurons, there is strong evidence supporting a direct and causal relationship between HCRT neurons that are stimulated by prenatal exposure to rewarding substances and the reward-motivated behavior observed early in life in the offspring. The stimulatory effects of prenatal exposure to various rewarding substances on the neurogenesis, expression and density of HCRT neurons in the embryo and after birth in postnatal and adolescent offspring are invariably accompanied by and positively related to changes in reward-motivated and emotional behavior [211,218,254]. These behavioral changes include an increase in the consumption of alcohol, nicotine and a fat-rich diet and of alcohol and nicotine co-use and also in anxiety and hyperactivity that are often associated with the intake of rewarding substances. In addition to stimulating HCRT neurons in their normal LH location, prenatal alcohol exposure induces ectopic HCRT neurons in regions anterior to the hypothalamus, the nucleus accumbens and caudate putamen, which mediate behaviors related to alcohol drinking, and these ectopic neurons shown in newborn offspring are still detected in early adolescent rodents. Furthermore, embryonic exposure to alcohol increases the number of processes that emanate from the normally located HCRT neurons, and it stimulates the long projections of the anterior ectopically expressed HCRT neurons, causing them to project to further anterior forebrain areas that are not normally innervated but involved in promoting reward-motivated behavior [218,238]. Together, these studies suggest that HCRT neurons similar to MCH neurons have an important role in promoting the reward-motivated and emotional behavior stimulated in the offspring by prenatal alcohol exposure.

### 7.5. Relation of Embryonic Substance-Induced Effects on HCRT Neurons to Early Behavioral Disturbances in Young Zebrafish

With zebrafish being particularly useful for studies involving direct manipulations of the peptide neurons in the LH, investigations using this animal model to study HCRT neurons provide direct support for their role in mediating drug-induced behavioral disturbances, perhaps through their close interaction with MCH neurons. This is demonstrated by evidence showing that the behaviors, including an increase in alcohol consumption produced at an early age by embryonic alcohol exposure [218,310,322,323], are similarly induced by direct optogenetic stimulation of the HCRT neurons [305,324]. These behaviors are also blocked by laser ablation of these HCRT neurons [218], consistent with other rodent studies showing similar behaviors to be increased by the chemogenetic as well as optogenetic activation of HCRT neurons [50,325,326].

Examination in zebrafish of the projections of these HCRT neurons after embryonic alcohol exposure provides further evidence for the involvement of specific neurons, particularly those projecting to anterior brain regions, in mediating disturbances in reward-motivated behavior [218]. These anterior HCRT neurons, which normally innervate the subpallium that contains HCRT receptors and is homologous to the mammalian basal ganglia and rich in DA [56,327,328], develop longer anterior projections after alcohol exposure which innervate a new structure, the dorsal pallium, that also participates in drug responses [329,330]. These neurons do not project to the hindbrain regions innervated by posterior HCRT neurons unaffected by alcohol, including the locus coeruleus and raphe nucleus known to mediate the sleep–wake cycle and arousal [331,332] and are suggested to be involved in the sleep disorders often comorbid with SUDs [333,334]. Direct support for the role of these anterior-projecting ectopic HCRT neurons in mediating the alcohol-induced disturbances in reward-motivated behavior is provided by evidence that the laser ablation of these specific HCRT neurons blocks the behavioral effects caused by embryonic alcohol exposure, including an increase in locomotor and anxiety behavior [218]. Further support for their role in behavior comes from the finding that these alcohol-stimulated anterior neurons have a distinct characteristic of densely expressing HCRT but exhibiting no expression of DYN [239]. With DYN shown to be negatively related to the drinking of alcohol [335,336] and to participate in the aversive effects of nicotine [337,338] and cocaine [339], these HCRT neurons that lack DYN are likely to be more effective in stimulating the consumption of alcohol or other rewarding substances.

### 7.6. Relation of Embryonic Substance-Induced Effects on Intracellular Molecular Systems to Disturbances in Behavior

Molecular systems such as transcription factors, growth factors and chemokines that are expressed in the MCH and HCRT peptide neurons and stimulated by prenatal exposure to reinforcing substances are likely involved in mediating the substance-induced increase in reward-motivated and emotional behavior in the offspring. A role of the transcription factor PPARβ/δ that is expressed and stimulated in the peptide neurons by prenatal exposure to a fat-rich diet [73] is suggested by evidence in rodents that PPAR isoforms are involved in the overeating of this diet and the excess consumption of rewarding drugs induced by exposure to a fat-rich diet [79,80,81]. It is also supported by evidence that PPARs are stimulated by prenatal exposure to nicotine, which stimulates MCH neurons and reward-seeking behavior [211,250], and by clinical and animal reports showing PPAR isoforms to affect both the positive and negative reinforcing properties of alcohol, nicotine and cocaine in adults [82,340,341].

Studies of the FGF2/FGFR1 system that is also expressed and stimulated in MCH neurons suggest the involvement of this growth factor system in mediating the behavioral effects produced in the offspring by prenatal exposure to rewarding substances. This is supported by evidence in rodents that maternal administration of FGF2 has stimulatory effects in the offspring on MCH neurons and behavior strikingly similar to those produced by prenatal exposure to alcohol, and maternal administration of an FGFR1 antagonist or FGF2 antibody blocks these alcohol-induced neuronal and behavioral changes in the offspring [92]. Further, FGF2 is stimulated by prenatal exposure to such substances as alcohol [92] and by neonatal exposure to nicotine [253], and there is evidence that FGF2 has an important role in regulating alcohol drinking [91,93,94,95] and the self-administration of cocaine [96,97,342]. This function of FGF2 in stimulating the development of MCH neurons and the reward-driven behavior it promotes may additionally involve the actions of transcription factors such as PPARβ/δ, which is shown to have a modulatory effect on growth factors [74,343].

The CCL2/CCR2 chemokine system that is expressed in MCH neurons [109,110] is also involved in mediating the behavioral changes induced by prenatal exposure to rewarding substances. This chemokine system, like MCH and HCRT, is stimulated in rodents by prenatal alcohol exposure, and it is positively linked to the overconsumption of rewarding substances like alcohol and a high-fat diet [121,122,123,124], to cocaine-induced locomotor sensitization [125] and to behaviors such as anxiety and locomotor activity [126,127,128]. Also, the behavioral effects including an increase in alcohol drinking and peptide neurons induced in the offspring by prenatal alcohol exposure are mimicked both by maternal administration of CCL2 and by CCL2 administration directly into the embryo brain, and they are blocked by prenatal administration of a CCR2 antagonist and CCL2 neutralizing antibody [109,110]. The consumption of a high-fat diet is also reduced by a genetic deficiency of CCR2 [134].

The CXCL12/CXCR4 system, which is more heavily expressed in HCRT than MCH neurons [111], may also be involved in mediating the behavioral as well as neuronal effects induced by prenatal exposure to rewarding substances. This is suggested by a variety of evidence in rodents, showing that endogenous CXCR4 expression is positively associated with behaviors like locomotor activity and depression that are linked to excess consummatory behavior [138]; maternal administration of CXCL12 increases anxiety in the offspring that is often associated with alcohol drinking [135]; and CXCL12 injection into the third ventricle stimulates the consumption of a palatable high-fat diet [134]. Moreover, the administration of a CXCR4 receptor antagonist is found to block cocaine-induced increase in conditioned place preference and locomotor activity [137]. In zebrafish, the overexpression of CXCL12a is shown to have stimulatory effects similar to embryonic alcohol exposure on behavior, while increasing the number of anterior and ectopic HCRT neurons and the length and density of their long anterior projections [117]. Further, these behavioral as well as neural effects of alcohol are blocked by the knockdown of CXCL12a or administration of a CXCR4 antagonist, supporting a direct role of this specific chemokine system in mediating alcohol’s stimulatory effects on early signs of reward-seeking behavior as well as embryonic development of the HCRT system [117,129].

### 7.7. Relation of Embryonic Substance-Induced Effects on DA Neurotransmission to Changes in Peptide Neurons and Behavior

While exposure to rewarding substances in adults and adolescents and the MCH and HCRT peptide neurons themselves are shown to have a strong stimulatory effect on the DA system that mediates reinforcement, studies of the effects on DA produced by prenatal exposure to rewarding substances reveal a very different effect. Multiple studies demonstrate that prenatal exposure to rewarding drugs and palatable food causes a strong and consistent suppression of the DA system throughout the offspring brain. This suppressive effect on DA in the midbrain where their neurons are concentrated is observed in rodent offspring prenatally exposed to alcohol [344,345,346], nicotine [347,348], cocaine [349,350,351,352] and a fat-rich diet [292,353] and also in zebrafish embryonically exposed to alcohol [354]. This suppression of DA neurotransmission may be related to the substance-induced stimulatory effects on the peptide neurons that stimulate DA and reward. The reduced functioning of the DA system is suggested to produce a marked deficit in reward which, in turn, recruits multiple brain systems that increase the drive to consume these substances and restore homeostasis within the reward circuit [355,356,357]. The evidence that MCH and HCRT neurons are both stimulated by prenatal exposure to rewarding substances suggests that they are a component of the neurocircuit that is activated to help compensate for this reward deficit and restore reward homeostasis, both by stimulating DA and increasing the motivation to consume rewarding substances. The alcohol-stimulated HCRT neurons that lack DYN, which normally provides a negative feedback signal to reduce drug-induced DA release and reward-seeking behavior [358], appear to be especially designed to perform this function.

### 7.8. Conclusions

The evidence described here, obtained using different methods as summarized in Figure 4, suggests that the stimulatory effects of embryonic exposure to rewarding substances on MCH and HCRT neurons in the hypothalamus and their intracellular molecular systems are involved in and possibly causally related to the behavioral changes that also occur early in life, including the overconsumption of rewarding substances and the various emotional behaviors that accompanies their abuse.

## 8. Sex Differences in Effects of Rewarding Substances on the Peptide and Molecular Systems in Relation to Behavior

While sex differences in any brain or behavioral system are difficult to characterize and mixed results are often obtained in clinical and animal studies, there is some evidence revealing sex differences in the specific hypothalamic neuropeptide/molecular systems and reward-motivated and emotional behavior examined in this report and the stimulatory effects produced by continued exposure to rewarding substances. A comprehensive review of the literature consistently shows that females are more strongly affected than males. Also, the sex difference similarly seen for the substance-induced changes in both the brain and behavior provides further support for the close relationship described above that exists between substance-induced neuronal changes in the embryo and behavioral disturbances observed at a young age.

### 8.1. Sex Differences in the Expression and Functions of MCH and HCRT Neurons and Their Intracellular Molecular Systems

There are studies in adults showing that the stimulatory effect of MCH on eating behavior has sexually dimorphic properties [5,359,360], with a close relationship to female steroids [361,362,363], and females exhibit a greater sensitivity than males to disturbances in MCH signaling following the consumption of nicotine [22]. In addition, examination of MCH neurons and their projections reveals sex differences, with transient expression detected in females but not males in the MCH neurons of reproduction-related subregions like the medial preoptic area and hypothalamic paraventricular nucleus, particularly during lactation [364], and with MCH neuronal circuits to adipose tissue shown in females to project both to retroperitoneal and subcutaneous fat tissue but only to inguinal white adipose tissue in males [365]. The HCRT system that interacts closely with MCH neurons also has sexually dimorphic properties in mediating the development of emotional conditions. This is demonstrated by the significantly greater changes in endogenous HCRT in female patients compared to males having an increased risk for depression [366], dementia [367] and Alzheimer’s disease [368]. Further, the DA system that is stimulated by both MCH and HCRT is expressed and exerts effects in a sex-dependent manner, and it is suggested to have a role in mediating the sex differences in SUDs involving the overconsumption of alcohol and nicotine [369,370,371,372]. Sex is also an important factor in the various functions of the molecular systems that are expressed in peptide neurons and involved in the control of consummatory behavior. Females compared to males exhibit stronger adaptive neuroimmune responses to PPAR agonists [373,374], have higher levels of circulating FGF2 [375] and show resistance to the inhibitory effect that an FGFR1 antagonist has on alcohol consumption [91]. Also, a positive relation to estrogen is shown for each of these molecular systems, including PPARγ [376,377], FGF2 [378] and chemokines [379].

### 8.2. Sex Differences in the Effects of Substance Exposure in Adults on Reward-Motivated and Emotional Behavior

While studies are limited, there is some evidence that adult exposure to or consumption of rewarding substances alters the reward-motivated behavior, peptide neurons and their molecular systems in a sex-dependent manner, with females more consistently affected than males. Clinical studies examining the development of SUDs demonstrate that females exhibit a faster progression from first use to the onset of alcohol use disorder and daily smoking [380,381]. They are also more vulnerable than men to such drugs as alcohol and niotine [382,383] and have more severe functional impairments with internalizing neurological conditions such as anxiety, depression and eating disorders more commonly diagnosed in women [384,385,386]. These clinical findings are consistent with studies in adult rodents, with females shown to consume higher amounts than males of drugs such as alcohol, nicotine and cocaine [387,388,389] and more likely to acquire drug self-administration and reinstate drug-seeking behaviors [387,389,390,391]. Also in rodents, alcohol or nicotine intake during adolescence produces a greater consumption in females as they become adults [392,393], and females have a higher preference for palatable fat-rich food [394], metabolize nicotine at a higher rate [395,396] and exhibit a higher expression of HCRT neurons in the LH with respect to drugs such as cocaine [397,398]. Moreover, the inflammatory neuroimmune pathway exhibits strong sex differences in response to various drugs. Females show a greater vulnerability to the neurotoxic and negative consequences of chronic alcohol drinking and have higher levels of proinflammatory chemokines and other inflammatory mediators known to be stimulated by estrogen [379,399,400]. The effects of tobacco smoke on inflammation in humans and cocaine in rodents are also sex-related [401,402], and females tend to exhibit greater neuroimmune responses than males to the consumption of a fat-rich diet [403].

### 8.3. Sex Differences in the Effects of Embryonic Substance Exposure on MCH and HCRT Peptide Neurons in the Offspring

Whereas clinical studies have yielded mixed results for sex differences in the behavioral effects of prenatal exposure to rewarding substances often due to various confounding experimental factors such as the timing of exposure, dose and method of self-reporting [404,405,406], there are some reports revealing sex-dependent effects, with female offspring after prenatal exposure exhibiting greater changes in reward-motivated behavior. Clinical reports examining children prenatally exposed to alcohol and studying behaviors related to neurological disorders including SUDs demonstrate that female progeny compared to male progeny have a greater probability of developing fetal alcohol syndrome disorder and higher rates of anxiety and depressive/mood, and they exhibit a greater increase in alcohol drinking, cigarette smoking and illicit drug use in adolescence and adulthood [406,407,408,409,410]. These behavioral effects of prenatal exposure to rewarding substances are accompanied by sex differences in brain development, with the female offspring shown to exhibit greater brain dysmorphology and a smaller grey matter volume than male offspring [406,409,410,411]. There is also evidence that prenatal exposure to nicotine leads to sex-dependent effects in the offspring, causing in female offspring thinner brain regions in the cerebral cortex, a greater vulnerability to developing nicotine dependence and an increase in nicotine consumption, with the male offspring more likely to develop attention deficit hyperactivity disorder [404,412,413,414]. Prenatal cocaine exposure also produces sex-dependent effects in offspring, with girls compared to boys showing greater anxiety in response to stress [415], more behavioral problems including internalizing behavior [416] and a greater sensitivity to the negative effects of cocaine exposure on self-regulatory functioning [417].

Studies in rodents and zebrafish of embryonic exposure to rewarding substances similarly reveal sex differences, showing a greater vulnerability of female than male offspring to their effects on both the brain and behavior. Prenatal exposure to alcohol at low doses demonstrates in females a significantly larger increase in the genesis as well as the expression and density of MCH neurons in the LH of postnatal and adolescent offspring [109,110] and also in the early development of MCH neurons from progenitor cells in the neuroepithelium of the embryo [109,111,216]. The effects of prenatal alcohol exposure on HCRT neurons are similarly sex-dependent, with female postnatal offspring exhibiting a greater increase in the expression and density of HCRT neurons compared to male offspring [111]. Sex differences are also evident in the brain of larval zebrafish, with embryonic exposure to alcohol at a low dose causing in females but not males a significant increase in the density of hypothalamic HCRT neurons [237]. These sex-dependent effects on the peptide neurons are associated in both rodents and zebrafish with similar sex differences in the stimulatory behavioral effects mediated by these peptides, supporting a close relationship between the changes in the brain and behavior. Females from an early age through adulthood exhibit after prenatal alcohol exposure a greater increase than males in the voluntary consumption of alcohol and in emotional/neurological behaviors including anxiety, locomotor activity and novelty-induced freezing behavior [110,237] and impulsive behavior and responses to stress or drug challenges [409,418,419]. While the effects of prenatal exposure to nicotine on MCH neurons and behavior have been examined only in male offspring [211,232] and sex differences related to prenatal cocaine have yet to be described, there are reports in rodents showing sex differences in the effects that prenatal exposure to a fat-rich diet has on measures of alpha-melanocyte stimulating hormone fiber density in adolescents [420] and of hippocampal neurogenesis and the endocannabinoid system in newborn offspring [421].

### 8.4. Sex Differences in the Effects of Embryonic Substance Exposure on Molecular Systems in Peptide Neurons of the Offspring

In addition to the changes in MCH and HCRT peptide neurons, embryonic exposure to alcohol has stimulatory effects on their intracellular molecular systems, including FGF2 and the CCL2 and CXCL12 chemokines and their receptors, that are consistently sex-dependent and significantly stronger in females than males. Studies in rodents demonstrate that the prenatal alcohol-induced increase in FGF2 mRNA levels in the LH occurs in females but not males, while the stimulatory effect on FGFR1 mRNA in the LH is similarly evident in females and males [92] as shown for this receptor in the periphery [375,422,423]. Furthermore, the stimulatory effect of prenatal alcohol exposure on MCH neurons co-expressing FGF2 also occurs only in females [92], an effect most likely mediated by estrogen that is stimulated by FGF2 [378], positively regulates FGF2-induced activation in endothelial cell proliferation [424] and is elevated by prenatal alcohol exposure [425,426], and the stimulatory effect on MCH neurons co-expressing FGFR1 transcripts is once again shown to occur similarly in both sexes [92]. The possibility that the FGF2/FGFR1 system mediates the sexually dimorphic effects of prenatal substance exposure on the development of peptide neurons is supported by the sex-dependent distribution of these transcripts within individual MCH neurons after prenatal alcohol exposure, which increases the number of FGF2 and FGFR1 transcripts in the nucleus as well as the cytoplasm of neurons in females but increases these transcripts only in the cytoplasm of males [92]. Together with evidence that FGF2 functions specifically within the nucleus to stimulate the proliferation and differentiation of different cell types [88,89,90], this finding suggests that this FGF2/FGFR2 system may have an important role particularly in females in mediating the stimulatory effect of prenatal alcohol exposure on both the MCH neurons and reward-motivated behavior.

The stimulatory effects of prenatal alcohol exposure on the proinflammatory chemokine systems in MCH neurons are also sex-dependent, consistently stronger in female than male offspring or not even detected in males. These effects are seen with the examination of CCL2 and CCR2 mRNA that is co-expressed with MCH in LH neurons and in radial glia neuroprogenitor cells in the hypothalamic neuroepithelium of the embryo [109,110,216]. Whereas a sexually dimorphic expression of CCL2 is not evident when levels are low under basal conditions as shown in the hypothalamus [109,110,216] as well as the hippocampus and cortex at any age [427], the stronger prenatal alcohol-induced increase in CCL2 levels in the LH that occurs in females suggests that this sex difference is challenge-based, consistent with measurements of CCL2 in the cortex [428]. It is also cell-type-dependent, with the alcohol-induced stimulatory effect on CCL2 in the LH shown to be sex-dependent and stronger in females than males for the large CCL2 neurons that mostly co-express MCH but not for the small CCL2 neurons that surround MCH neurons [216]. An important role of the CCL2/CCR2 system in mediating the sexually dimorphic nature of the prenatal alcohol-induced effects on MCH neurons receives direct support from evidence that the stimulatory effects produced by maternal administration of CCL2, like alcohol, are also sex-dependent and far greater in female offspring, which show an unusually large increase in CCR2 expression and a greater increase in alcohol consumption, effects that are blocked by a CCR2 antagonist [110,216]. A rodent study involving the in utero administration of CCL2 directly into the embryonic brain on embryonic day 14 when neurogenesis peaks reveals similar sex-dependent effects, with female but not male embryos exhibiting a significant increase in endogenous CCL2 within radial glia cells and their processes in the neuroepithelium that branch through the medial hypothalamus toward the LH [118]. These results in the brain are consistent with the behavioral evidence showing that deletion of the CCL2 gene reduces alcohol consumption in females but not in males [121]. While also showing no sex difference under control conditions, the increased expression produced by prenatal alcohol exposure in rodents on CXCL12 and CXCR4 in MCH neurons of the LH and in radial glia neuroprogenitor cells of the embryonic neuroepithelium is strongly sex-dependent, consistently greater in female than male offspring [111]. With other evidence showing the expression of an estrogen and its receptors to be stimulated by alcohol [425,429,430] and positively linked to the chemokine system [379,399,400], the alcohol-induced upregulation of HCRT neurons that occurs more strongly in females may involve this steroid receptor, a role likely to be indirect with no studies yet showing the HCRT neurons to express this receptor.

### 8.5. Conclusions

The results summarized here consistently show sex differences, stronger in females than males, not only in the functions of MCH and HCRT neurons and their molecular systems but also in their responses to the stimulatory effects of the rewarding substances. These sex differences are shown with adult and adolescent exposure as summarized in Figure 2; embryonic exposure to these substances on the development of these peptide and molecular systems in the embryo as shown in Figure 3; and the behavioral disturbances that accompany these changes in neural systems as described in Figure 4. These consistent sex differences provide further support for a close and possibly causal relationship between substance-induced effects on the brain and disturbances in behavior.

## 9. Overall Conclusions and Future Direction

The clinical and animal studies summarized in this review support the idea that MCH neurons in the hypothalamus have an important role in mediating reward-motivated consummatory and emotional behavior. These neurons with projections to limbic/striatal forebrain regions mediate this behavior through positive interactions with the DA system; the HCRT neurons that are anatomically and functionally linked to MCH neurons; and the intracellular molecular systems like PPAR, FGF2 and chemokines that stimulate the development and migration of these peptide neurons. We present here evidence that exposure in adults and adolescents and even during embryonic development to rewarding substances, including commonly used drugs such as alcohol, nicotine and cocaine and palatable fat-rich food, stimulates the expression of these neuropeptide and molecular systems in the hypothalamus and increases reward-seeking and related emotional behavior, revealing a positive feedback loop that leads to the overconsumption and abuse of these substances and the development of neurological conditions as described in clinical studies (Figure 2). Models involving embryonic exposure to these rewarding substances that allow more in-depth analyses in animals of the embryo and young offspring greatly advance our understanding of how the rewarding substances affect the development of these neuropeptide/molecular systems and mediate this positive feedback loop (Figure 3). These studies using different methods also demonstrate how these changes in the brain systems contribute to the various disturbances and their sex differences in reward-driven and emotional behavior early in life that increase the risk of developing neurological disorders later in life, during adolescence and in adulthood (Figure 4).

Of particular note is that the behavioral changes produced across animal species by exposure to rewarding substances even at low levels are similarly observed in clinical studies. This suggests that the same neuropeptide/molecular systems shown in animals to be stimulated and involved in producing behavioral disturbances may also be functional in humans. Furthermore, as in animal studies, clinical studies demonstrate that females are at a greater risk than males for developing SUDs with co-occurring emotional disorders and female offspring exhibit stronger adverse responses than male offspring to maternal consumption of the rewarding substances during pregnancy. In addition, both clinical and animal studies demonstrate that exposure to rewarding substances at any stage of life is a strong predictor of an individual’s vulnerability to develop neurological disorders such as SUDs, especially if this exposure occurs during pregnancy when the brain is developing and most sensitive to environmental influences including commonly used drugs and palatable food.

The evidence described here strongly and directly relates substance-induced changes in the neuropeptide/molecular systems to disturbances in behavior, a relationship that deserves further attention. With the rise in recent years of recreational drug use through vaping by teenagers and the availability of highly palatable prepared foods occurring widely throughout the world, there is an urgent need to address the lack of knowledge and raise awareness in the young population and their parents about the serious and harmful effects produced by early excess exposure to these rewarding substances. This education along with research to develop new therapeutic behavioral strategies can help them to carefully weigh the risks and benefits of excess drug use and diet intake, make informed decisions and set realistic goals themselves as to when and how often to consume these rewarding substances.
